# Using Intervention Mapping to Codevelop Orchid, a Digital Tool for Reproductive Life Planning: Development and Feasibility Study

**DOI:** 10.2196/87650

**Published:** 2026-07-02

**Authors:** Catherine Stewart, Helen Carr, Maitri Shila Tursini, Alice Howe, Jennifer Hall

**Affiliations:** 1 Reproductive Health Research Department, Institute for Women’s Health, University College London, 74 Huntley Street, London, England, United Kingdom, 44 20 7679 2000; 2 NHS Surrey Heartlands Health and Care Partnership, Guildford, United Kingdom; 3 Homerton University Hospital NHS Foundation Trust, London, England, United Kingdom

**Keywords:** digital health intervention, reproductive life plan, reproductive health, preconception health, preconception care, contraception, intervention mapping

## Abstract

**Background:**

Most people make no health or lifestyle changes before pregnancy, missing a key opportunity to improve outcomes. Consequently, nearly half of UK pregnancies are unplanned, disproportionately affecting underserved groups and widening health inequalities. Digital health interventions (DHIs) offer promise but require systematic, theory-driven development to ensure effectiveness and real-world applicability.

**Objective:**

This study aimed to use Intervention Mapping to codevelop and pilot Orchid, a novel DHI designed to support people of reproductive age to understand their pregnancy preferences and develop a reproductive life plan (RLP).

**Methods:**

We used Intervention Mapping steps 1‐4 to guide the systematic, theory-informed codevelopment of Orchid. A multidisciplinary planning group and codevelopment group of 21 members of the public contributed throughout. At step 1, previous research and a scoping review of existing RLPs informed the program goals and logic model of the problem. During step 2, we identified performance objectives and behavioral determinants to specify practical strategies for each target behavior. At step 3, we applied the Capability, Opportunity, Motivation–Behavior (COM-B) model and relevant behavior change techniques to guide intervention design. Finally, at step 4, Orchid was co-designed as a website and mobile app providing users with a pregnancy preference group and prediction of pregnancy, a dynamic RLP, tailored evidence-based information, and optional goal-setting features to support behavior change. Orchid was piloted between January and May 2025 to explore its feasibility and acceptability in health care settings. Interviews with users, nonusers, and health care professionals were conducted and quantitative data from users were collected.

**Results:**

These findings indicate that implementation was feasible, and health care professionals found it acceptable to recommend Orchid to patients, though noted barriers including time constraints and competing priorities. Overall, 153 people signed up to Orchid; 68% (72/106) of eligible users received a pregnancy preference group and 27% (32/119) of eligible users completed a full RLP. Users were positive about Orchid, appreciating its content and design, noting that Orchid contained a wealth of information about reproductive health presented in an easy-to-understand manner. They valued the autonomy, convenience, and privacy afforded by the digital format, and found it acceptable to be recommended Orchid within a health care setting. Orchid uptake was lower than anticipated, and use was limited; this was partly expected given the short pilot period, but feedback also suggested targeted recruitment and navigation improvements could enhance uptake and engagement.

**Conclusions:**

Orchid is the first co-designed DHI to support reproductive health across the life course. Its systematic development, theoretical foundation, strong user involvement, and positive pilot testing position it as a promising, scalable innovation to support reproductive health, deliver credible information in accessible formats, and promote preventative, community-based care across the National Health Service.

## Introduction

### Background

Within the United Kingdom, nearly half of all pregnancies are unplanned [1], and abortion rates are at an all-time high [2]. These trends suggest that reproductive health services are failing to meet people’s needs. Contraceptive use remains inconsistent, with almost 1 in 5 women of reproductive age in England reporting using no contraception or unreliable methods [3], and the use of hormonal contraception is decreasing [4]. Unplanned pregnancies are disproportionately concentrated among underserved populations [5], including those living in poverty and individuals from ethnic minority groups, and are associated with a range of adverse outcomes such as preterm birth, involvement of children’s social care services, financial adversity, and health inequalities [6]. As such, unplanned pregnancy remains a significant public health concern and a key driver of disparities in maternal and child health.

At the same time, the health of individuals entering into pregnancy (whether planned or unplanned) presents additional challenges. More than 90% of women in the United Kingdom have at least one modifiable risk factor associated with adverse pregnancy outcomes [7] including maternal and neonatal morbidity and mortality. Yet, the majority of women, and their partners, make no health or lifestyle changes in preparation for pregnancy [8]. This lack of planning and preparation represents a missed opportunity to optimize health before conception, improve pregnancy outcomes, and reduce longer-term risks for both parent and child. Contributing to this gap is the fact that discussions around pregnancy preferences and preconception health are not routine in health and social care settings [9,10], even for people with preexisting conditions, those taking medications, or those with social risk factors.

Furthermore, reproductive health discourse and interventions often overlook the role of men, despite evidence linking paternal nutrition, body composition, and lifestyle behaviors to sperm quality and offspring health outcomes [11,12]. This underscores the need for a more inclusive, holistic approach to reproductive health.

There is an unmet and expressed need among people of reproductive age for better support with their reproductive health [9,13]. Combining this need with the challenges outlined above highlights the importance of adopting a more proactive, person-centered approach to supporting reproductive decision-making. Reproductive life planning [14] offers such an approach, enabling individuals to reflect on their reproductive intentions, align these with their health and life circumstances, and access the information and care needed to achieve their goals.

While health care professionals have a vital role to play in delivering this support, additional approaches are needed to extend reach and accessibility. Digital health interventions (DHIs) offer a promising approach, with emerging evidence suggesting that they can overcome barriers of distance, accessibility, stigma, and judgment [15] and can improve preconception health [16]. However, evidence on their effectiveness in supporting contraception use remains limited [17,18].

The development of any new DHI should follow a systematic, theory-driven process to maximize its potential for effective real-world implementation. In line with Medical Research Council guidance on complex interventions [19,20], such development should be reported transparently to strengthen the evidence base, enhance reproducibility, and allow others to assess its relevance across contexts. Despite these imperatives, the intervention development phase remains relatively underreported, which poses issues with transparency, quality, and user-centeredness [21].

### Aim

This paper aims to describe the systematic development and pilot testing of Orchid, a novel DHI to support people of reproductive age to understand their pregnancy preferences and develop a reproductive life plan (RLP).

## Methods

### Development of Orchid Using Intervention Mapping Framework

Our development of Orchid followed the Intervention Mapping (IM) framework [22], as used by other digital health programs [23].

#### Step 1: Needs Assessment

##### Establish and Work With a Planning Group

Our planning group, that met weekly, was comprised of 2 female researchers (JH and CS) who had led the development work underpinning Orchid, 3 female clinicians (HC and MST [2 general practitioners] and AH [sexual and reproductive health doctor]) affiliated with the team, and a male software developer.

While previous work was focused on women, early input from the codevelopment group highlighted that including men would be important both to reduce the disproportionate burden placed on women and to address the underrepresentation of men in reproductive health research. Therefore, we created a codevelopment group of 21 public members (males and females, aged 16‐45 y, with diverse ethnicities, relationship statuses, sexuality, and family sizes, living across the United Kingdom). Members were recruited from existing Public and Patient Involvement groups and the school where Orchid was piloted.

##### Conduct Mixed Methods Needs Assessment

We conducted primary research on the feasibility and acceptability of different ways of assessing pregnancy preferences, followed by a scoping review, to explore the existing evidence on RLPs [24], to identify key goals for Orchid.

##### Primary Research

The Pregnancy Planning, Preparation, and Prevention Study (P3 Study) explored ways of assessing women’s feelings and preferences regarding a future pregnancy [25]. Within its cohort, 80% of women expressed a preference for exploring their pregnancy preferences digitally [9]. This preference was consistent across different age groups and was further validated through qualitative research involving in-depth discussions with women [13] and reflects findings from other studies [26]. Qualitative work with health care professionals [13] (HCPs) reinforced that a digital tool providing accessible reproductive health information could usefully extend support beyond clinical encounters. Although HCPs viewed discussions about pregnancy preferences as acceptable and valuable, many reported lacking the confidence, training, and time to deliver them effectively [13].

The P3 study also evaluated the psychometric performance and predictive validity of the UK version of the Desire to Avoid Pregnancy (DAP) scale [27] and found it to be a valid and reliable measure of women’s desire to avoid pregnancy, as well as highly predictive of pregnancy within the next 12 months (sensitivity 78% and specificity 81% [27,28]).

##### Scoping Review

We carried out a scoping review of the published and gray literature to map the evidence on RLPs [24]. Most studies and gray literature originate from the United States, primarily within clinical settings. Across the literature, 21 different RLPs were identified, with many based on the 2006 Moos RLP [29]. Digital RLPs were scarce, with none currently available in the United Kingdom.

Acceptability of RLPs was consistently high across studies; users were overwhelmingly positive about being asked about reproductive life planning. However, evidence of effectiveness was limited. There was minimal evidence of the application of behavior change theory in the development of the RLPs, with only 3 tools (all digital) describing the inclusion of specific behavior change theories [30-32].

##### Identify Program Goals

The needs assessment helped to identify the program goals: to create a digital tool (app and website) to help people of reproductive age understand their pregnancy preferences, support them to develop and implement an RLP, and increase awareness of reproductive health. The aim is to support users to achieve their reproductive health goals, reduce unintended pregnancies, increase pregnancy preparations, and empower decision-making.

##### Create a Logic Model of the Problem

We created a logic model (Table S1 in Multimedia Appendix 1) of the problem from the findings of our scoping review and previous research.

### Step 2: Selecting Behavioral Determinants and Performance Objectives

Informed by the results of our needs assessment and input from our codevelopment group, we identified specific performance objectives for subpopulations, based on reproductive preferences. For example, taking folic acid for those actively planning a pregnancy or consistent and correct contraceptive use for those wishing to avoid pregnancy. See Table S2 in Multimedia Appendix 1 for more examples.

Our scoping review identified 5 key behavioral determinants that are underlying drivers of behavior change: (1) knowledge [30], (2) self-efficacy [31,32], (3) social support [31], (4) intrinsic motivation [30], and (5) autonomy [30]. We explored the relationships between performance objectives and behavioral determinants and identified change objectives and practical strategies for each target behavior.

### Step 3: Identifying Theory-Based Intervention Methods and Practical Applications

We applied the Capability, Opportunity, Motivation–Behavior (COM-B) model [33] when developing Orchid. This model was selected because each of the behavioral determinants we had identified (knowledge, self-efficacy, social support, intrinsic motivation, and autonomy) aligns with one or more of the COM-B components, providing a comprehensive framework for influencing behavior change within Orchid. We systematically mapped each component of the COM-B model to Orchid’s proposed content and features, linking them to the specific objectives identified during the planning phase (Table 1). This ensured that theory directly informed Orchid’s design, aligning practical features with the mechanisms expected to support behavior change. We also identified several behavior change techniques [34-37], including goal setting and reminders, feedback, habit stacking, action planning, information, and education. These techniques were incorporated into Orchid to support the COM-B framework, enhance engagement, and increase the likelihood of successful behavior change.

**Table 1.** Mapping the COM-B^a^ model components as applied to Orchid against the preconception and contraception pathways.

Orchid intervention	COM-B components	Link to behavior change
Preconception		
Evidence-based questionnaire or risk assessment	Psychological capability Reflective motivation	Builds awareness and knowledge of health status. Prompts self-reflection and understanding of risk factors that may need addressing.
Informational content on health optimization	Psychological capability Reflective motivation	Enhances users’ knowledge and skills to make informed decisions. Supports internal motivation by linking actions to outcomes.
Access to relevant resources	Physical opportunity Psychological capability	Enhances opportunity by increasing access to information.
Find your nearest health care provider or support groups	Social opportunity Physical opportunity	Enhances opportunity by increasing access to services and connecting users to support systems.
Goals and reminders for preconception behaviors	Reflective motivation Automatic motivation	Supports planning and habit formation. Reminders and prompts maintain engagement and drive action.
Positive reinforcement messaging	Automatic motivation	Builds positive associations, rewards behavior, and sustains engagement over time.
Contraception		
Personalized contraceptive questionnaire	Psychological capability Reflective motivation	Builds understanding of contraceptive choices and helps users make informed decisions that fit their lifestyle, increasing likelihood of consistent use.
Educational content on contraception	Psychological capability Reflective motivation	Enhances knowledge, increasing competence and confidence to choose and use contraception effectively.
Access to relevant resources	Physical opportunity Psychological capability	Increases access to credible information and reduces barriers.
Goals and reminders for contraceptive use	Automatic motivation Reflective motivation	Encourages habit formation and planning, reducing forgetfulness, and promoting consistent use.
Action planning for contraceptive scenarios	Psychological capability Reflective motivation	Builds self-regulation and coping strategies, increasing users’ ability to maintain contraceptive use across variable contexts.
Find your nearest health care provider	Physical opportunity Social opportunity	Improves access to professional support and normalizes help-seeking behavior.

a COM-B: Capability, Opportunity, Motivation–Behavior model

### Step 4: Program Development and Pilot Testing

#### Development of Orchid Prototype

Between November 2022 and February 2023, we codeveloped a prototype of Orchid. This incorporated the DAP scale and a digitized pregnancy prediction algorithm [28] to present users with a pregnancy preference group, their likelihood of pregnancy, and tailored resources. Throughout the process, our codevelopment group emphasized the importance of ensuring that Orchid is inclusive of all users, including men, and expressed a desire for Orchid to be available as both an app and a website to enhance accessibility.

#### Development of Orchid Version 1

**Figure 1.**
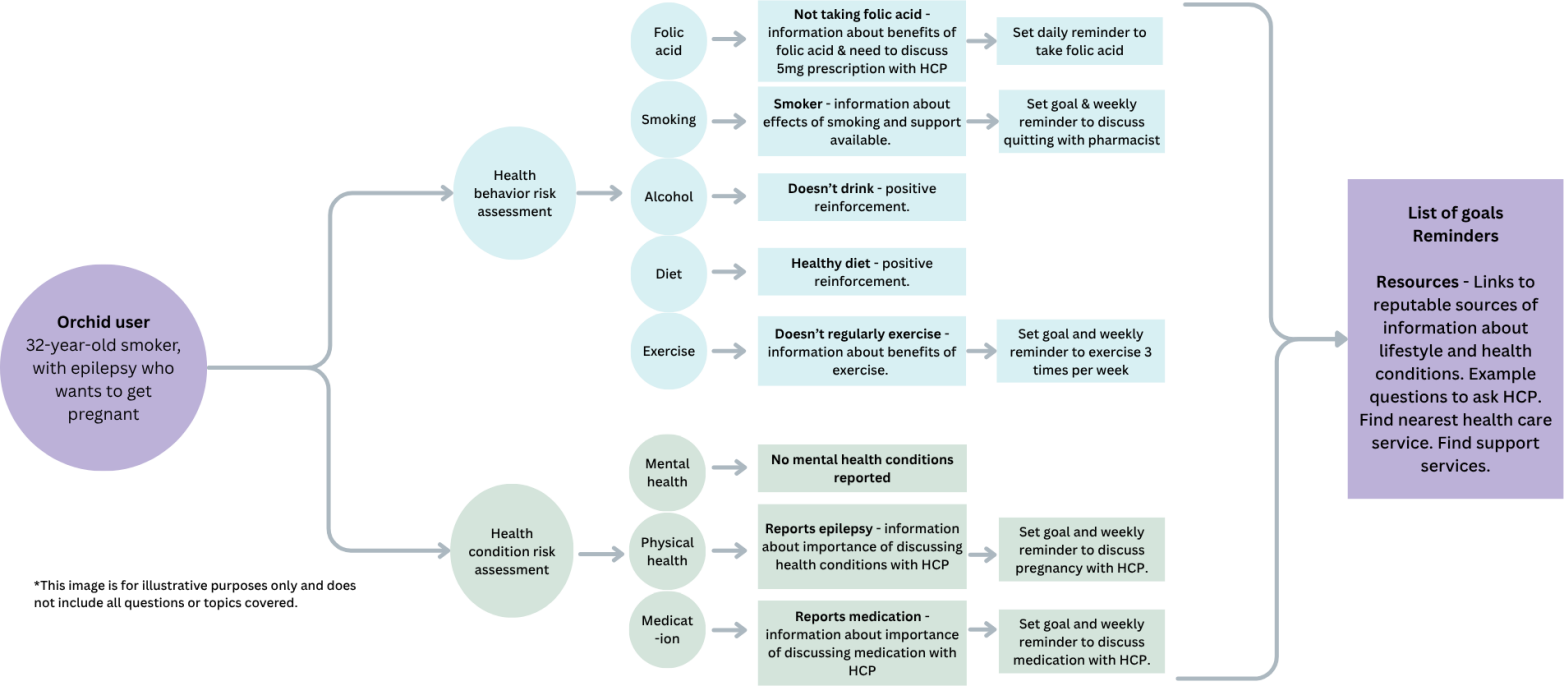
Walk-through of Orchid preconception Reproductive Life Plan pathway. HCP: health care professional.

Starting with the Orchid prototype and guided by the COM-B model, program goals, and aims, we worked collaboratively with our program team and codevelopment group to build the RLP component of Orchid (Figure 1). Our public contributors worked with us throughout to enhance accessibility and engagement. They played a central role in shaping the content and user pathways; their input led to substantial expansion of the scope to encompass sexual health, menstrual health, menopause, cancer awareness, and decision-making. The codevelopment of Orchid was an iterative process using a mixture of online group meetings (n=3) and surveys (n=10) from February to December 2024.

Users are required to sign in to Orchid to protect their data and ensure confidentiality. They then answer a series of questions about their reproductive preferences (DAP Scale) [38]. Based on their answers, users are assigned a pregnancy preference group (eg, Planner, Preventer, and Pauser) and given a personalized pregnancy prediction. This prediction is generated by an algorithm (developed by JH) that combines DAP responses with selected sociodemographic characteristics to calculate the likelihood of becoming pregnant in the next year [39]. Full details on pregnancy preference group classification and the pregnancy prediction algorithm are provided in the Multimedia Appendix 1.

**Figure 2.**
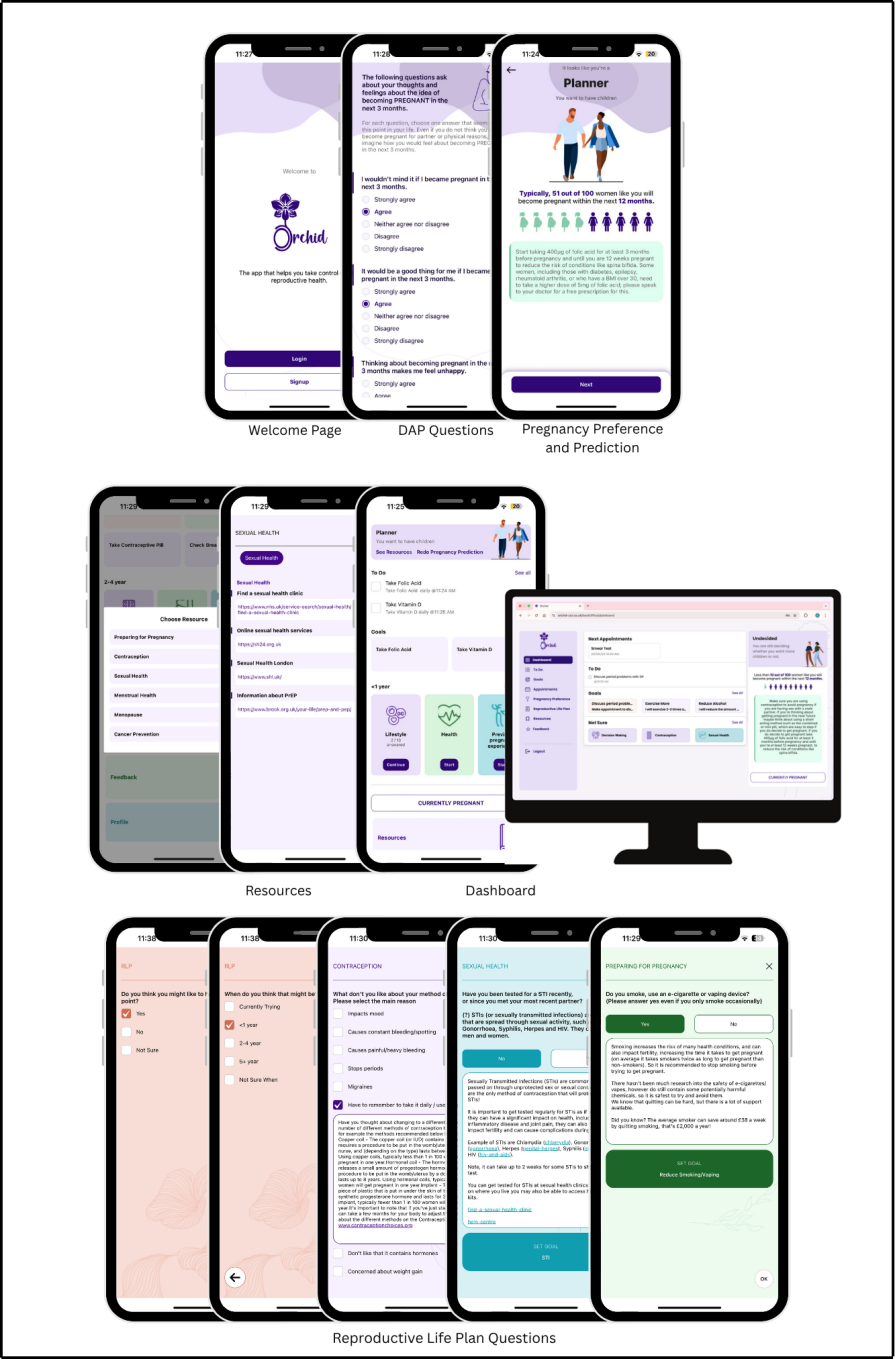
Orchid overview. DAP: Desire to Avoid Pregnancy scale.

Users are then guided down a dynamic pathway of questions that adapts based on their answers to create a comprehensive RLP. Relevant, evidence-based information is provided along the pathway and signposted in the Resources section. As users move down the pathway, they can set optional to-do items and reminders, aimed at assisting users to take action to achieve their goals. For details, see Figure 2 for an overview of Orchid.

#### Pilot Testing to Explore Acceptability and Feasibility

We conducted a pilot between January and May 2025 to explore the acceptability and feasibility of implementing Orchid in health care and educational settings. We collaborated with our codevelopment group to develop the ethics application for this study, codeveloping recruitment materials, such as posters and SMS text messages, participant information sheets, topic guides, and lesson plans. This paper focuses on the health care pilot; the school pilot is detailed separately [40].

#### Health Care Pilot

We piloted Orchid in 3 National Health Service (NHS) health care settings in Greater London: a GP practice, a sexual health clinic, and a women’s health hub (WHH). The pilot ran for 5 months with GP and sexual health clinic sites launching in January 2025, followed by the WHH in February 2025. All study locations concluded activity at the end of May 2025.

Each site received a 1-hour training session from a clinician prior to the launch of Orchid, covering how and why to ask about pregnancy preferences, the principles of reproductive life planning and preconception health, and an introduction to Orchid and the pilot study. The content and style of the training program were based on Normalization Process Theory (NPT) [41,42] and COM-B [33]. The NPT constructs of context, mechanisms, and outcomes were considered, particularly aiming to present a coherent message that was cognitively engaging and included suggestions for collective action in that context. A presentation was adapted for each training site and an electronic copy of the slides was provided afterward. We delivered the training in person at 2 sites and online at the third. In total, 36 HCPs attended the training.

Patients (aged 18‐50 y) were invited to use Orchid through 3 methods: text messages sent via Accurx (used only at the GP site), posters displayed in the waiting rooms (GP and sexual health clinic), and HCP recommendations during clinical consultations (all sites).

At the GP practice (the only site with a defined patient list), text messaging served as the primary recruitment method. Overall, 4456 eligible patients were identified and contacted in 2 batches: the first half received an initial invitation on January 8, 2025, and the second half on January 16, 2025. All patients were recontacted on February 7, 2025, followed by a targeted reminder sent on April 30, 2025, to 939 patients who had attended an appointment in the previous 2 months. In contrast, precise numbers of eligible patients could not be determined for the sexual health clinic or WHH, as neither holds a registered patient list. However, the sexual health clinic sees approximately 60‐80 patients per day, while the WHH sees around 20‐30 patients per week (with many ineligible for Orchid due to age criteria).

#### Orchid User Quantitative Data

Within Orchid, we collected quantitative data on uptake, completion of Orchid, sociodemographics of users, recruitment method, and location. Completion was defined as reaching the end of a personalized RLP pathway. Users were grouped according to their pregnancy preferences as follows: Planner (wants a pregnancy now), Undecided (has not decided whether they want children), Postponer (wants children in the future), Pauser (has at least 1 child and would like another in the future), Finisher (has all the children they want), and Preventer (does not want children). Quantitative data were analyzed using Stata/MP 18 (StataCorp).

#### Health Care Professional Interviews

HCPs from each of the 3 participating sites were recruited via email. To be eligible for an interview, HCPs were required to have attended the Orchid training.

#### Orchid User and Nonuser Interviews

We recruited Orchid users and nonusers through separate strategies to ensure representation of both groups. All registered Orchid users were invited to participate via email. Information about interviews was also included on posters displayed at sites. Nonusers were identified and invited through GP-sent Accurx text messages; patients received an initial message asking whether they had accessed Orchid, and those who responded “no” were asked about their reasons for nonuse and invited to take part in an interview. For details, see Figure S1 in Multimedia Appendix 1 for a flow diagram outlining the participant recruitment process.

All interviews were conducted via Zoom (Zoom Communications, Inc), audio-recorded with participant consent, and transcribed verbatim by a member of the research team. Following data familiarization, we collaboratively developed a shared coding framework for the user and nonuser interviews through group discussions, drawing on our research questions and early impressions from the transcripts. Using this common framework, user interviews were coded by CS and nonuser interviews by JH. In parallel, HCP interviews were analyzed using a separate coding framework informed by NPT [41], with coding undertaken by HC. To ensure analytic consistency, the full team met regularly to discuss emerging codes, compare interpretations, and refine frameworks as needed. These meetings supported iterative theme development and enabled us to explore differences and commonalities across user, nonuser, and HCP accounts, ensuring that all final themes were firmly grounded in the data. Qualitative data were analyzed in NVivo 14 (Lumivero).

Qualitative data collection concluded once all users, nonusers, and eligible HCPs who responded and consented had been interviewed.

### Ethical Considerations

Ethical approval for the health care pilot was granted by the NHS London, Riverside Research Ethics Committee (Ref: 24/LO/0421). Informed consent was obtained from all Orchid users; they were provided with a participant information sheet and were required to provide consent before using Orchid. Informed consent was also obtained from all users, nonusers, and HCPs who participated in an interview. Participants who took part in interviews were given a £25 (US $33) voucher.

All user data collected through Orchid were handled in accordance with data protection regulations. Email addresses were stored separately from the main dataset, and all analytical data were pseudonymized prior to analysis. All data were stored in the University College London Data Safe Haven and were only accessible to authorized members of the research team.

## Results

All data presented are new and unique to this manuscript.

### Health Care Professional Interviews

We conducted in-depth interviews with 10 HCPs. The NPT [41] framework was used to analyze the data, exploring the effectiveness of the training, the likelihood of adoption, implementation, and sustainment of Orchid. Outcome data were limited by the short-term nature of the pilot project, and we therefore focused on the intervention performance.

Training was feasible in all 3 clinical settings and was scheduled during each site’s regular training program. In-person sessions were preferred to live online training, but HCPs agreed that online was sometimes more practical. Most HCPs found the training stimulating and understood the importance of the topic. New learning and increased confidence were commonly reported, and HCPs appreciated receiving detailed practical tips that they could immediately incorporate into their clinical practice. The inclusion of men in the RLP message was novel for most and several specifically commented on their cognitive engagement with the training, saying,

*I thought the training was great, because it actually got me thinking differently.*

[CC05]

Most HCPs said that being able to use Orchid within the training would have increased their confidence in sharing the tool with patients:

[MP02]

Despite this, most HCPs found signposting patients to Orchid acceptable, although some demonstrated limited engagement; one HCP said,

*I’ve discussed it with some patients, but I haven’t found any leaflets... I’m kind of forgetting … about it*.

[CC05]

Many HCPs felt that reminders or brief refresher sessions would be useful:

*Just like a little nudge for people sometimes … just kind of brings it back up to the top of your memory*.

[CC02]

and emphasized the need for more collective action to support sustained uptake of Orchid. Having a local champion at a site was seen as valuable in maintaining momentum, as illustrated by one HCP:

*So [name] … works in the clinic, I know she just gets [the leaflets] out and puts them on the desk as well, so, so everybody hopefully knows about it and brings it up*.

[WHH02]

HCPs found it feasible to incorporate opportunistic RLP and preconception information into certain consultations, particularly those relating to women’s health, baby clinics, vaccination clinics, chronic disease clinics, and male fertility. One HCP noted

*It doesn’t take long … anything related to women’s health is really easy*.

[WHH01]

However, HCPs agreed that it was more challenging to introduce these topics in other consultations:

*It’s just difficult to talk contraception when somebody comes for something completely different*.

[WHH01]

and many acknowledged that such discussions were not always possible:

*You have your agenda of keeping the patient safe, and they have their agenda of what they want to get sorted out, and then this is like another thing to add into the mix*.

[MP04]

Overall, views were mixed on the impact on consultation times; while some HCPs were hesitant to raise the topic for fear of it leading to a longer consultation, others acknowledged that Orchid could “save time maybe because … we can refer directly to the app and say, look, we have good information there” (MP01).

Importantly, where HCPs had mentioned Orchid to patients, the response was consistently positive:

*I have recommended Orchid to a few patients, and they’ve been really interested in it*.

[WHH02]

At the GP practice, text messages were used to promote Orchid. While HCPs were supportive of this method of recruitment, “I think the text message is absolutely excellent,” they did share concerns about potential pitfalls:

*Sometimes I do get patients worrying that the texts they’re getting are scams and lots of people don’t speak … or can’t read English.*

[MP04]

### Orchid Users’ Quantitative Data

In total, 153 people signed up to Orchid, of whom 109 (71%) were females and 44 (29%) were males. Most people found out about Orchid via a text message from their GP (102/153, 66.6%), followed by clinical consultation (19/153, 12.4%) and posters in waiting rooms (15/153, 9.8%). The most common reason for using Orchid was “I want reliable information” (48/153, 31.3%), followed by “just curious” (36/158, 23.5%). Sociodemographic data of Orchid users can be seen in Table 2.

**Table 2.** Orchid users’ sociodemographic characteristics.

Demographic	Values
Age (n=136), mean (range)	33 (18‐50)
Sex (n=153), n (%)	
Female	109 (71.2)
Male	44 (28.8)
Ethnicity (n=145), n (%)	
White	59 (40.7)
Black, Black British, Caribbean, or African	37 (25.5)
Asian or Asian British	18 (12.4)
Mixed or multiple ethnic groups	14 (9.7)
Other ethnic group	17 (11.7)
Relationship status (n=146), n (%)	
Married / or civil partnership	47 (32.2)
In a relationship, living with partner	23 (14.8)
In a relationship, not living with partner	36 (24.7)
Single	34 (23.3)
Other	6 (3.7)
Sexual orientation (n=142), n (%)	
Heterosexual	108 (76.1)
Bisexual	16 (11.3)
Gay or lesbian	10 (7.0)
Other	8 (5.6)
Education (n=135), n (%)	
Degree or above	76 (56.3)
Diploma in HE^a^	10 (7.4)
A Level or College	30 (22.2)
GCSE^b^	14 (10.4)
None	5 (3.7)
Employment (n=143), n (%)	
Employed full or part time	93 (65.0)
Looking after home or family	9 (6.3)
Long-term sick leave or disabled	11 (7.7)
Student, in full-time education	17 (11.9)
Unemployed	11 (7.7)
Other	2 (1.4)
English main language (n=153), n (%)	
Yes	117 (76.5)
No	36 (23.5)
Index of multiple deprivation quintile (n=102), n (%)	
1 (Most deprived 20% of areas)	47 (46.1)
2	44 (43.1)
3	6 (5.9)
4	2 (2.0)
5 (Least deprived 20% of areas)	3 (2.9)

a HE: higher education.

b GCSE: General Certificate of Secondary Education.

Of the 106 nonpregnant female users, 72 (68%) received a pregnancy preference group (Table 3). Of the 119 people who were eligible to complete an RLP pathway (3 pregnant females, 72 females who received a pregnancy preference group, and 44 males), 32 (26.9%) did so (Table 3). There was no difference in completion by ethnicity. More details of who was eligible for the different features can be found in the Multimedia Appendix 1.

**Table 3.** Percentage of Orchid users in each pregnancy preference group and Reproductive Life Plan pathway.

Groups	Orchid users in each group, n (%)
Pregnancy preference group^a^ (n=72)	
Planner	26 (36.1)
Undecided	24 (33.3)
Postponer	11 (15.3)
Pauser	5 (6.9)
Finisher	4 (5.6)
Preventer	2 (2.8)
Reproductive Life Plan pathway^b^ (n=66)	
Currently trying	7 (10.6)
1 year	12 (18.2)
2‐4 years	8 (12.1)
5+ years	3 (4.6)
Not sure when	6 (9.1)
Not sure if	13 (19.7)
Don’t want	11 (16.6)
Currently pregnant or expecting	6 (9.1)

a Pregnancy preference groups: Planner (wants a pregnancy now), Undecided (has not decided whether they want children), Postponer (wants children in the future), Pauser (has at least one child and would like another in the future), Finisher (has all the children they want), and Preventer (does not want children).

b Reproductive Life Plan pathways: users who are currently pregnant or expecting, currently trying to conceive, users who would like to have a child within 1 year, within 2‐4 years, or in 5+ years, users who want a child but are unsure when, are unsure if they want children, or do not want (more) children.

### User and Nonuser Interviews

In total, 5 users and 2 nonusers were interviewed, and we also received written feedback (via Accurx text messaging) from 2 additional nonusers.

#### Background of Users and Nonusers Interviewed

A total of 4 users interviewed were female and one was male. Users ranged in age from 22 to 35 years, and none had children, although all expressed a desire to have them in the future. Several users had experience with reproductive health conditions, including polycystic ovary syndrome, irregular menstrual cycles, and fibroids. The 2 nonusers interviewed were both female and aged 40‐49 years; 1 had children and did not want any more, while the other had never been pregnant and did not want children. We have no sociodemographic information on the 2 nonusers who provided written feedback via text message.

#### Digital Reproductive Health Information

While most users felt confident discussing reproductive health with HCPs, they noted that clinicians often lacked the time or sometimes the specific knowledge needed to answer all questions or provide tailored advice. As a result, users liked the idea of receiving information about reproductive health digitally and felt that digital tools such as Orchid would be a way of overcoming some of these barriers. Users viewed digital tools as a direct and convenient method of accessing information and found that digital content was often more personalized. Users also felt that digital tools would allow *“*much more productive conversations with a health professional*”* (U05), as it would help *“*answer … questions before … seeing a doctor … and then know … what I’m looking for from the doctor*”* (U04).

#### Orchid Recruitment

The primary method of recruitment for the interviewed users was through text messages sent by their GP, with most agreeing that this was an effective approach as receiving the message directly from their GP made it feel official and trustworthy.

Most users chose to visit or download Orchid because they were intrigued by the tool’s focus on providing information about reproductive health, with some expressing particular interest due to personal circumstances, such as wanting to learn more about preparing for pregnancy or managing reproductive health conditions. However, 1 user and 3 of the nonusers thought that Orchid was primarily for “women who are wanting to be pregnant” (U02), with the nonusers citing this as the reason why they had not used Orchid, as pregnancy was not something they currently wanted.

#### Orchid Use

Users were divided on whether they accessed Orchid through the app or the website. Website users typically did so because they were unaware that an app was available or preferred to explore the web version before committing to downloading the app.

Ongoing use of Orchid was limited, with most users reporting that they accessed it only once or twice, primarily to complete the questions and review relevant information. None of the users engaged with the goal or reminder features within the app, citing that goals were either not relevant to their current needs or they were unaware that they could set them. Some users did follow links to external “useful resources” [U05] to find out more information about topics discussed within Orchid. Users also indicated that they would return to the app in the future, particularly if their circumstances changed (eg, if they began actively trying for a pregnancy) and would then be more likely to use features such as goal setting and reminders.

Despite limited use, users reported learning new information from Orchid, particularly regarding preparing for pregnancy. For example, one user reported that Orchid had “chang[ed] my outlook on planning” (U04), while another noted that they had begun taking supplements because of the information provided in Orchid.

#### Orchid Feedback

Overall, users were positive about Orchid, appreciating both its content and design. They noted that the app contained a wealth of useful information about reproductive health, presented in an easy-to-understand manner. Users also praised the app’s look and feel, describing the design as nice and simple, “well laid out” (U05) and “easy to navigate” (U01). They valued the adaptive flow of questions, which personalizes the experience based on users’ answers, and appreciated the ability to *“*go back up the path and say … I’ve changed my mind*”* (U02), allowing Orchid to adjust as their needs or decisions changed.

In addition to the positive feedback, users also identified several areas where Orchid could be improved. Many wanted more varied content, such as images and videos, as well as more comprehensive explanations and additional topics. Some users found navigating the web-based version on a mobile phone challenging, and users reported difficulty locating where to download the app. Despite these issues, all users said that they would recommend Orchid to friends and family.

## Discussion

### Principal Findings

This study set out to codevelop and pilot Orchid, a novel DHI, to support people of reproductive age to understand their pregnancy preferences and form an RLP. Guided by the IM framework [22], we codeveloped Orchid through iterative co-design with end users and a multidisciplinary clinical academic team. The pilot study, conducted across 3 NHS settings, demonstrated that integrating Orchid into real-world clinical contexts was feasible and acceptable. HCPs were generally comfortable recommending Orchid to patients, and users valued its content and design. Although uptake was lower than anticipated, the data collected provide important insights for refinements needed for wider implementation.

Using IM [22] enabled transparent, evidence-based codevelopment grounded in behavioral theory and user needs. The structured process supported iterative refinement and ensured alignment between Orchid’s purpose, content, delivery, and intended behavior change outcomes, avoiding assumption-led development. By mapping performance objectives and determinants to theory-based methods, grounding Orchid in COM-B [33], and incorporating behavioral determinants identified in the literature [24], we specified hypothesized mechanisms of action aligned with behavior change techniques shown to increase engagement [43]. This systematic approach enhances transparency and has been associated with improved DHI quality and effectiveness [44,45]. IM’s emphasis on needs assessment and stakeholder involvement further ensured that Orchid was relevant, inclusive, and user-centered, which was reflected in strong acceptability among health professionals and diverse users [46,47].

Implementation within health care settings proved feasible, with training integrating well into existing sessions and HCPs generally finding it acceptable to recommend Orchid. However, barriers emerged, including time constraints, competing clinical priorities, and difficulty raising reproductive health in unrelated consultations, which are common concerns [13,48,49]. Users found it acceptable to be recommended Orchid and valued its digital format due to the convenience, autonomy, and privacy it affords, consistent with previous findings [13].

Uptake was lower than expected, a common challenge in digital health research [50,51]. Feedback from those who received a GP text message but did not sign up uncovered a perception that Orchid was primarily for people planning a pregnancy, despite our efforts to use neutral “reproductive health” messaging. This highlights the need for tailored recruitment strategies that clearly communicate relevance, though evidence on personalized recruitment remains mixed [52]. Digital recruitment remains cost-effective [50], suggesting optimization is worthwhile [50]. HCPs valued the training but reported that the lack of a live demonstration limited their confidence in signposting, echoing wider evidence that successful DHI implementation requires practical exposure [53]. Addressing these factors is likely to improve recruitment in future roll-outs.

Engagement with Orchid during the pilot was limited, with infrequent use of goal-setting and reminder features. While partly expected due to the short pilot period, users reported barriers such as navigation challenges and not realizing Orchid could be downloaded as an app (where the goal-setting features are optimized). Despite this, users gained new knowledge and described ways in which Orchid informed their reproductive decision-making or led to behavior change, suggesting that even brief interventions can have a meaningful impact [54], as others have also found across a range of health conditions [51,55]. As part of IM step 4 [43], we will refine pathways through Orchid, reduce friction points, and expand features to support more sustained engagement, ahead of larger-scale evaluation (steps 5 and 6).

Existing RLP studies [56] demonstrate high acceptability but limited evidence of effectiveness [56], with most studies focusing on process outcomes, such as knowledge, rather than behavior change outcomes [24]. However, the OptimalMe study [57] found increased use of folic acid and reduced alcohol intake in women planning a pregnancy [57], while digital contraceptive interventions appear to be associated with increased uptake of effective contraception [58], especially post pregnancy, though this is not always the case [59]. As the first UK co-designed, theory-driven, digital RLP integrating reproductive preferences with both contraception and preconception pathways, Orchid fills longstanding gaps in scope, theory, and digital innovation.

As a feasibility and acceptability pilot, this study is limited. Key outcomes, including ongoing engagement, behavior change, and effectiveness, require evaluation in future stages. Additional limitations include the lack of sampling frames at 2 sites, preventing response rate calculation or assessment of generalizability, and uncertainty around exposure to different recruitment methods. The absence of a live app at the time of training may have reduced HCP signposting, and limited visibility of the app means we cannot draw conclusions about platform preference.

Orchid’s systematic development, theoretical foundation, and extensive end-user involvement represent key strengths commonly associated with effective DHIs [19,20,33,44,45,54]. Together, these features position Orchid well to function as an effective intervention for improving reproductive health [60]. Our emphasis on inclusivity is reflected in the diversity of both the codevelopment group and pilot users, including people from more deprived areas, speakers of languages other than English, global majority populations, men, and nonbinary people. The varied pregnancy preferences among users show that Orchid serves diverse needs. A particularly notable strength is Orchid’s explicit inclusion of men, a group often overlooked in preconception and reproductive life planning. The fact that nearly one-third of users were male highlights demand for male-inclusive reproductive health support and demonstrates Orchid’s reach among underserved groups, aligning with emerging evidence that systematically codeveloped DHIs demonstrate greater acceptability and effectiveness [19,20,33,44,45,54].

Orchid has been designed to support national priorities for digital transformation and preventative care, reduction of unplanned pregnancy, and improvements in maternity outcomes. The diversity of pilot users supports our aim of equitable reach. The NHS 10-Year Plan for England [61] prioritizes digital innovation, prevention, and strengthened primary care, and Orchid aligns with each of these aims. By offering accessible, evidence-based reproductive health information and supporting people to articulate and act on their reproductive intentions, Orchid has the potential to enhance digital health literacy, reduce service pressures, and improve patient-provider interactions. By addressing inconsistent contraception use, lack of proactive reproductive life planning, and low awareness of preconception health actions, Orchid can contribute to reducing unplanned pregnancy and improving maternal and neonatal outcomes, as well as longer-term health and development of parents and children.

### Conclusion

Orchid is the first DHI co-designed to support reproductive health across the life course in the United Kingdom. The development and pilot testing of Orchid demonstrate the value of using IM to create a theory-driven, codeveloped digital tool for reproductive health. The findings of our pilot suggest that Orchid is both feasible to implement and acceptable to health care providers and users, while highlighting areas for refinement to enhance engagement and relevance. Insights from the pilot will directly inform iterative improvements and the design of a larger-scale implementation evaluation study to evaluate effectiveness. With its evidence-based foundation, user-centered design, and alignment with national health priorities for prevention and digital transformation, Orchid holds promise as a scalable DHI to support reproductive decision-making, reduce unplanned pregnancy, and contribute to better maternal, parental, and child health outcomes across the NHS.

## Supplementary material

10.2196/87650 Multimedia Appendix 1 Orchid logic model, performance objectives and associated behavioral determinants, pregnancy preference group classifications, probability of pregnancy calculation, eligibility of Orchid features and Orchid recruitment flowchart.

## Abbreviations

COM-B: Capability, Opportunity, Motivation–Behavior model
DAP Scale: Desire to Avoid Pregnancy Scale
DHI: digital health intervention
GP: general practitioner
HCP: health care professional
IM: Intervention Mapping
MRC: Medical Research Council
NHS: National Health Service
NPT: Normalization Process Theory
RLP: Reproductive Life Plan
WHH: women's health hub
