# Supplementary material for: Using Intervention Mapping to Codevelop Orchid, a Digital Tool for Reproductive Life Planning: Development and Feasibility Study
Source: JMIR Hum Factors. 2026 Jul 2;13:e87650. doi: 10.2196/87650 (PMC13327371; doi:10.2196/87650)
Supplement: Multimedia Appendix 1 [file humanfactors-v13-e87650-s001.docx]

**Using Intervention Mapping to co-develop and pilot Orchid: a new digital tool for reproductive life planning.**

**Supplementary Materials**

**Authors**

Catherine Stewart^1^, MSc, 0000-0002-7204-7356.

Dr Helen Carr^1^,^2^, MBBS, 0009-0000-5958-6593.

Dr Maitri Shila Tursini^1^, MBBS., 0000-0001-6963-881X.

Dr Alice Howe^1,3^, MBChB, 0000-0001-6784-6551

Professor Jennifer Hall^1^, PhD, 0000-0002-2084-9568.

**Affiliations**

1. Reproductive Health, Institute for Women’s Health, University College London, London, UK.
2. NHS Surrey Heartlands Health and Care Partnership, Guildford, Surrey, UK.
3. Homerton Healthcare NHS Foundation Trust, London, UK.

| *Contents:* |  |
| --- | --- |
| *Table S1: Logic Model of the problem* | *2* |
| *Table S2: Examples of performance objectives and associated behavioural determinants for different sub-populations, defined by reproductive preferences and life stage* | *4* |
| *Table S3: DAP Score Classification to Pregnancy Preference Group.* | *5* |
| *Calculation of the predicted probability of pregnancy* | *6* |
| *Eligibility for Orchid Features* | *7* |
| *Figure S1: Orchid Recruitment Flow Chart* | *8* |

| **Inputs** | **Activities** | **Outputs** | **Outcomes** | **Impact** |
| --- | --- | --- | --- | --- |
| Development and maintenance of an app/web platform | **Pathway Design and Implementation**  -Create personalised user flows for each pathway using COM-B  -Develop content tailored to reproductive goals (e.g., conception, contraception, or sexual health, menstruation, menopause, cancer prevention, decision-making)  -Develop content for males, females, non-binary, trans. | Tailored user pathways addressing various reproductive intentions    Comprehensive content library covering conception, contraception, and pregnancy. | Improved decision-making in reproductive health    Better communication between couples wanting to get pregnant    Increased reproductive health awareness and literacy. | **Reduced unintended pregnancies** |
| Behavioural frameworks (COM-B) | **Integrate Behaviour Change Tools**  -Goal-setting features aligned with COM-B:  -Capability: Knowledge and skills development via educational resources.  -Opportunity: Accessible resources and reminders.  -Motivation: Visual goal progress and nudges. | Tools for reproductive health tracking and decision-making    Increased engagement with app and web platforms. | Increased planning for conception    Healthier lifestyle pre-pregnancy    Higher levels of satisfaction with current contraception    Reduced discontinuation of contraception | **Sustainable behaviour changes leading to healthier communities and intergenerational health** |
| Lived experience | **Co-development** with diverse group of people with lived experience | User insights integrated in iterative updates to pathways, content and recruitment methods | Enhanced uptake by underserved groups | **Reduced reproductive health inequalities** |
| Healthcare professionals | **Training** and development of educational resources | Increased knowledge and awareness of pregnancy intention screening and preconception health | Adoption in to routine practice | **Normalisation of reproductive life planning** |
| Teachers | Development of lesson plans and teacher **resources** | Increased teacher knowledge and confidence in teaching reproductive health content | Adoption in to education settings |  |
| Stakeholders | **Knowledge mobilisation** activities | Increased knowledge and awareness of the population benefits of pregnancy intention screening and preconception health | Incorporation in to policy and commissioining | **Improved population-level reproductive health outcomes** |

*Table S1: Logic Model of the problem.*

| **Sub-population** | **Performance Objectives** | **Behavioural Determinants** |
| --- | --- | --- |
| **Planning pregnancy** | Take folic acid daily | Knowledge of benefits / access to supplements / habits formation |
|  | Discuss health conditions/medications with HCP | Confidence to seek care / awareness of risks / trust in healthcare provider / access to care |
|  | Optimise preconception health (diet, smoking, alcohol, drugs, exercise) | Motivation / perceived importance / social support |
| **Preventing pregnancy** | Find a contraceptive method that suits preferences | Awareness of options / autonomy in decision-making / confidence in discussing choices / access to services and methods |
|  | Use contraception consistently and correctly | Knowledge of correct use / self-efficacy / access to contraception |
| **Postponing pregnancy** | Awareness of preconception health for future | Knowledge / risk perception / health literacy / motivation / self-efficacy |
|  | Continue effective contraception use | Habit formation / access to contraception / relationship dynamics / autonomy |
| **Undecided about pregnancy** | Reflect on life goals before having children | Future orientation / values clarification / social and cultural expectations |
|  | Consider practical aspects of parenthood | Financial awareness / perceived readiness / partner communication / social and cultural expectations |
| **Young people** | Understand consent and healthy relationships | Knowledge / social norms / social support / communication skills |
|  | Understand menstrual health | Access to information / confidence to discuss / social support |
|  | Seek medical advice for menstrual concerns | Confidence / motivation / reduced stigma / access to care |
| **Perimenopause / menopause** | Recognise symptoms and seek support if needed | Awareness / reduced stigma / motivation / access to care |
|  | Manage health risks (e.g., bone health) | Knowledge / perceived susceptibility / habits |
| **Cancer prevention** | Check breasts/testicles regularly | Knowledge of self-exam / motivation / perceive risk / habit formation. |

*Table S2: Examples of performance objectives and associated behavioural determinants for different sub-populations, defined by reproductive preferences and life stage.*

| **DAP Score + Socio-demographic Q** | **Pregnancy Preference Group** |
| --- | --- |
| DAP = <0.5 | Planner |
| DAP = 0.5–1 + Future children Y | Planner |
| DAP = 0.5–1 + Future children M | Undecided |
| DAP = 0.5–1 + Future children N | Undecided |
| DAP = >1–2 + Children N | Undecided |
| DAP = >1–2 + Children Y + Future children M | Undecided |
| DAP = >1–2 + Children Y + Future children N | Undecided |
| DAP = >1–2 + Children Y + Future children Y | Pauser |
| DAP = >2–3 + Future children M | Undecided |
| DAP= >2–3 + Children N + Future children N | Preventer |
| DAP = >2–3 + Children N + Future children Y | Preventer, for now |
| DAP = >2–3 + Children Y + Future children N | Finisher |
| DAP = >2–3 + Children Y + Future children Y | Pauser |
| DAP>3 + Children N + Future children N | Preventer |
| DAP>3 + Children N + Future children M | Undecided |
| DAP>3 + Children N + Future children Y | Preventer, for now |
| DAP>3 + Children Y + Future children M | Undecided |
| DAP >3 + Children Y + Future children Y | Pauser |
| DAP >3 + Children Y + Future children N | Finisher |

*Table S3: DAP Score Classification to Pregnancy Preference Group.*

*This table describes how participants were assigned to one of six pregnancy preference groups using their DAP score, in combination with responses to selected sociodemographic and reproductive history questions (including number of existing children and desire for future children). Group assignment was based on predefined scoring thresholds and decision rules.*

**Calculation of the predicted probability of pregnancy.**

Full details of the development of the multivariable model (algorithm) to provide the personalised prengnacy prediction are provided in reference 40: Hall JA, Barrett G, Stephenson J, Rocca CH, Edelman N. Predictive ability of the Desire to Avoid Pregnancy scale. Reprod Health. 2023 Sep 25;20(1):144. doi: 10.1186/s12978-023-01687-9. PMID: 37749640; PMCID: PMC10521409.

In brief, a multivariable logistic regression model was developed to estimate the odds of pregnancy within 12 months by including baseline socio‑demographic factors that were associated with pregnancy in univariate analyses and the DAP score. Age group, relationship status, number of children in the household, ethnicity, education level, and DAP score were initially entered into the model. In the fully adjusted analysis, relationship status, ethnicity, and education level were not independently associated with pregnancy and were therefore excluded, while age and number of children remained significant predictors. Within Orchid a person’s age, number of children and DAP score are used to provide a predicted probability of pregnancy based on this regression model.

**Eligibility for Orchid Features**

Within Orchid, only non‑pregnant female users are eligible to complete the DAP. This is because the DAP has only been validated in females and includes questions about future pregnancy preferences, making it inappropriate for individuals who are currently pregnant. Consequently, only non‑pregnant females can be assigned a pregnancy preference group based on their DAP results. Females who are currently pregnant do not complete the DAP and are automatically assigned to the “Currently Pregnant” group. Male users do not complete the DAP and are not assigned a pregnancy preference group. Instead, male and pregnant female users bypass the DAP and proceed directly to the RLP. Non‑pregnant female users become eligible to complete the RLP only after completing the DAP and receiving a pregnancy preference group.

Because features such as goal setting, to‑do lists, and reminders are available only within the RLP, access to these features is limited to users who initiate an RLP.


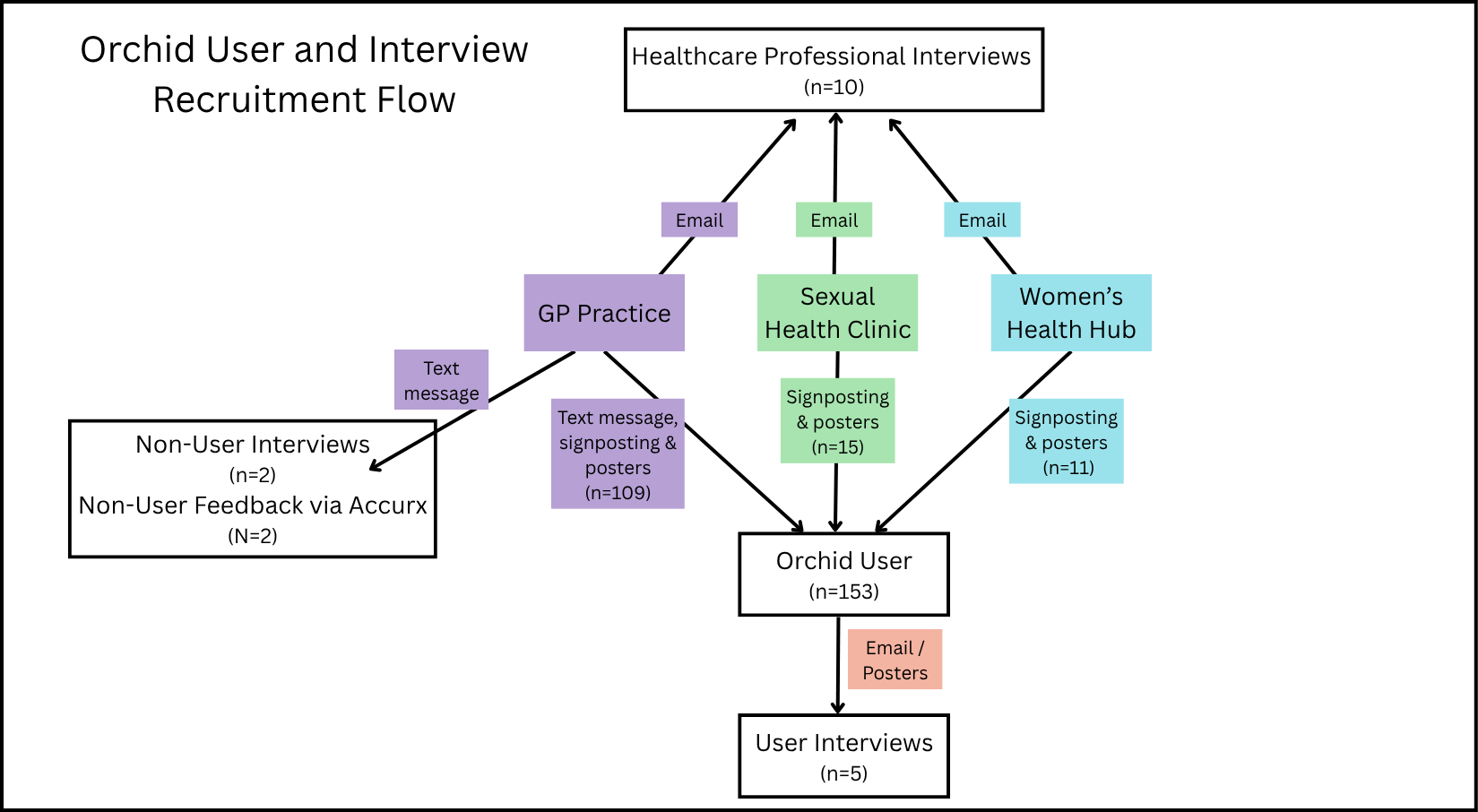
*Figure S1: Orchid Recruitment Flow Chart*
